# Supplementary material for: Mutant ASXL1 induces age-related expansion of phenotypic hematopoietic stem cells through activation of Akt/mTOR pathway
Source: Nat Commun. 2021 Mar 23;12:1826. doi: 10.1038/s41467-021-22053-y (PMC7988019; doi:10.1038/s41467-021-22053-y)
Supplement: Supplementary file 1 — Supplementary Information [file 41467_2021_22053_MOESM1_ESM.pdf]

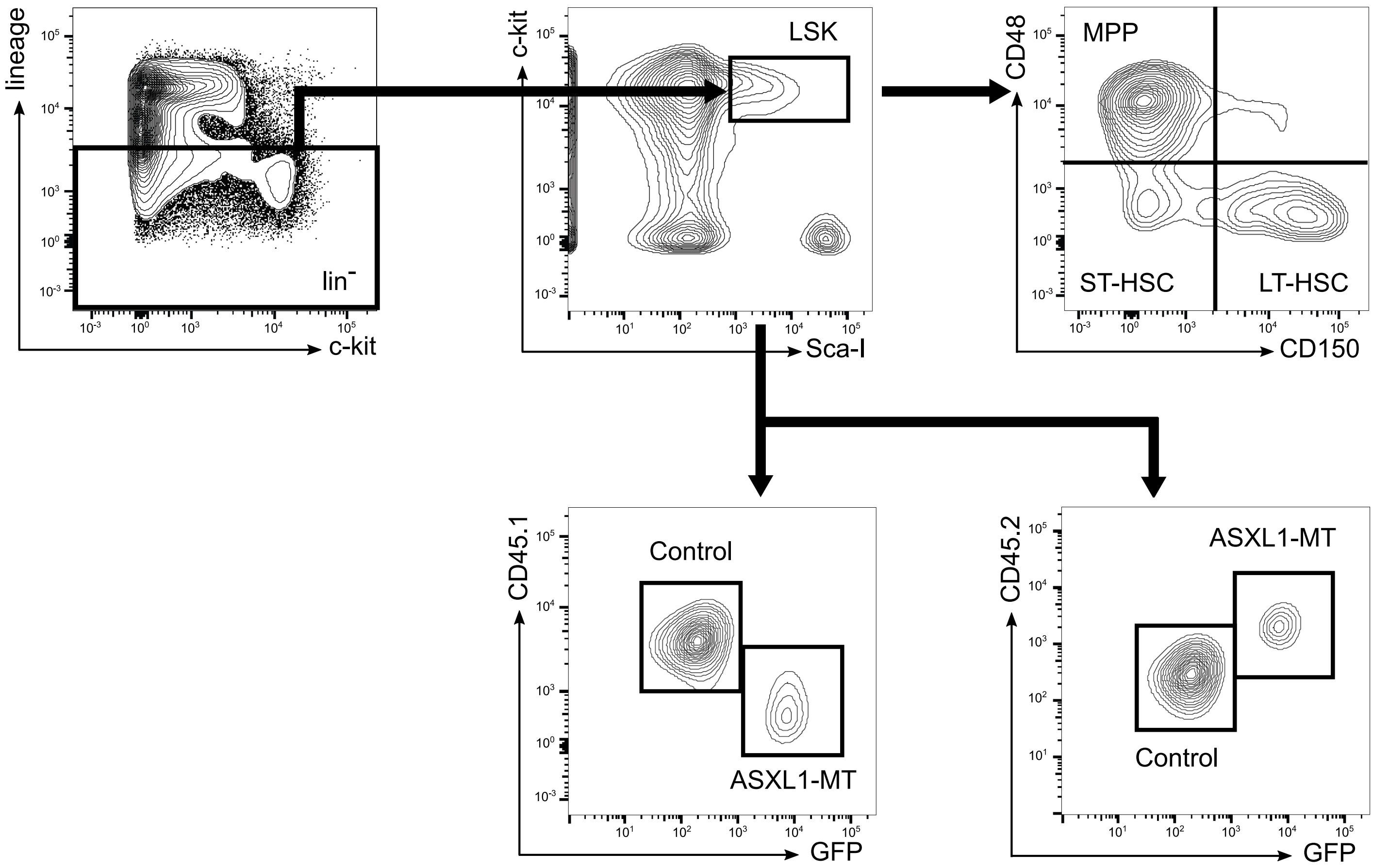

Supplementary Fig. 1

Supplementary Fig. 1 | Scheme of the flow cytometry analysis. Whole bone marrow cells isolated from control ($CD45.1^+$) and *Vav-Cre* ASXL1-MT KI ($CD45.2^+$) mice were transplanted into lethally irradiated recipient mice. As ASXL1-MT KI mice carry the floxed allele of ASXL1-MT-IRES-GFP, ASXL1-MT expressing cells are positive for both CD45.2 and GFP.

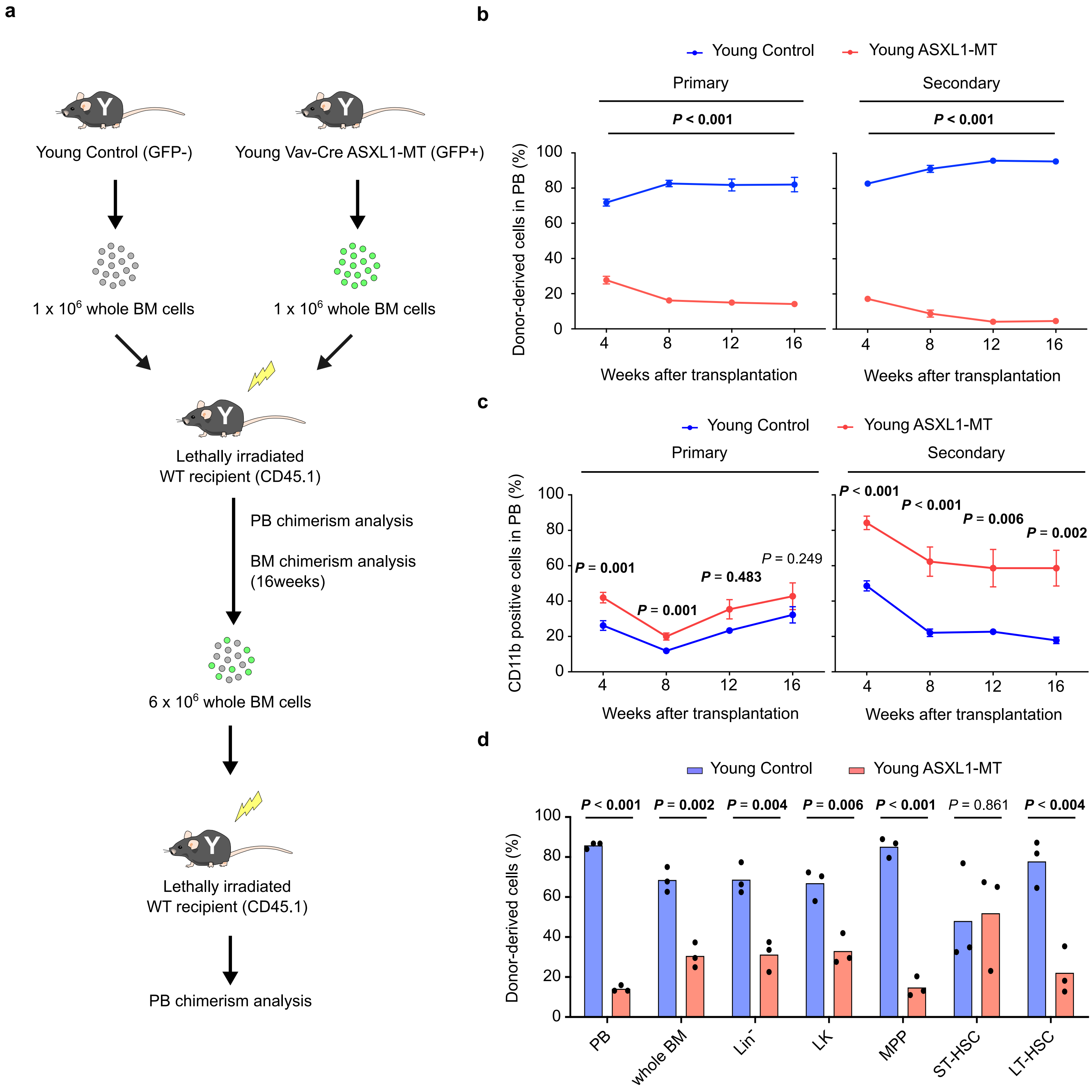

Supplementary Fig. 2

Supplementary Fig. 2 | ASXL1-MT impairs self-renewal of HSCs and promotes myeloid-biased differentiation after transplantation in young mice. a, The experimental design for serial transplantations. 1×10^6 bone marrow MNCs isolated from young control and young *Vav-Cre* ASXL1-MT KI mice were transplanted into lethally irradiated recipient mice (n=10). At 16 weeks after primary transplantation, secondary transplantation was performed using pooled 6×10^6 bone marrow MNCs from 3 primary recipient mice (n=8). **b,** Levels of donor chimerism in peripheral blood were analyzed at the indicated weeks after primary and secondary transplantations. **c,** The frequency of CD11b positive cells in peripheral blood at the indicated weeks after primary and secondary transplantations. **d,** 6 months after primary transplantation, the frequency of donor-derived cells in peripheral blood (PB), whole bone marrow cells, Lin^- cells, $Lin^-c\text{-}kit^+Sca1^-$(LK) cells, MPPs, ST-HSCs and LT-HSCs were analyzed (n=3). Data are mean ± s.e.m. **P*≤0.05, ***P*≤0.01, ****P*≤0.001; two-tailed Student's *t*-test.

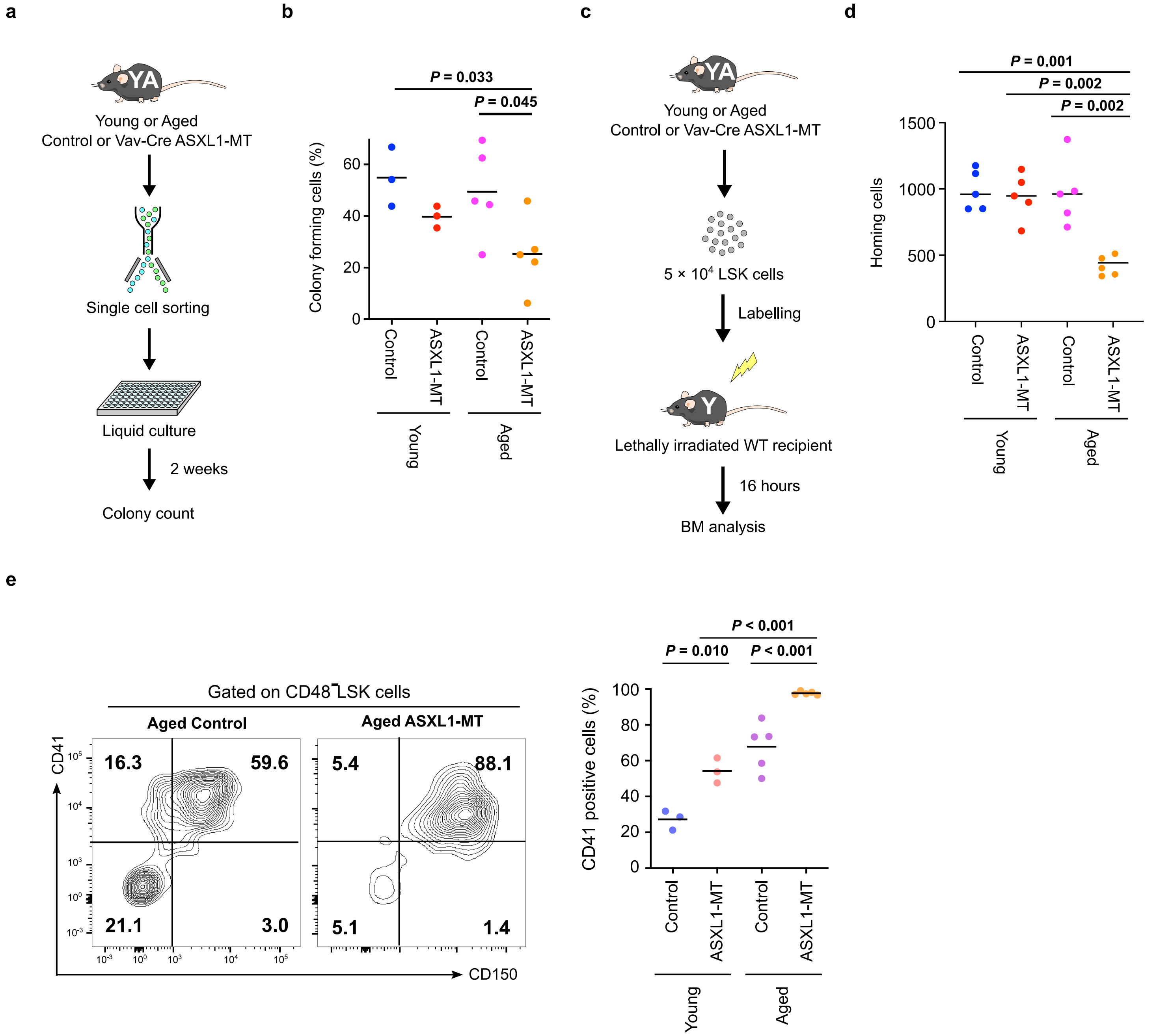

Supplementary Fig. 3

Supplementary Fig. 3 | The increased pLT-HSCs in aged *Vav-Cre* ASXL1-MT KI mice exhibit a functional decline. a, The experimental design for single cell liquid cultures. Single LT-HSCs isolated from young and aged *Vav-Cre* ASXL1-MT KI mice were cultured in liquid media for 2 weeks. **b**, The frequency of colony forming cells was measured (n=3 (Young) and 5 (Aged)). **c**, The experimental design for homing assays. 5×10^4 LSK cells isolated from young and aged *Vav-Cre* ASXL1-MT KI mice that had been stained with CellTrace dye were transplanted into lethally irradiated recipient mice. **d**, 16 hours after transplantation, bone marrow cells from recipient mice were analyzed. **e,** The frequency of CD41-positive cells in pLT-HSCs of young and aged *Vav-Cre* ASXL1-MT KI mice (n=3 (Control) and 5 (Aged)). Representative FACS plot (left panel) and summarized data (right panel) are shown. Data are assessed by one-way ANOVA with Tukey-Kramer's post-hoc test. *$P \leq 0.05$, **$P \leq 0.01$, ***$P \leq 0.001$.

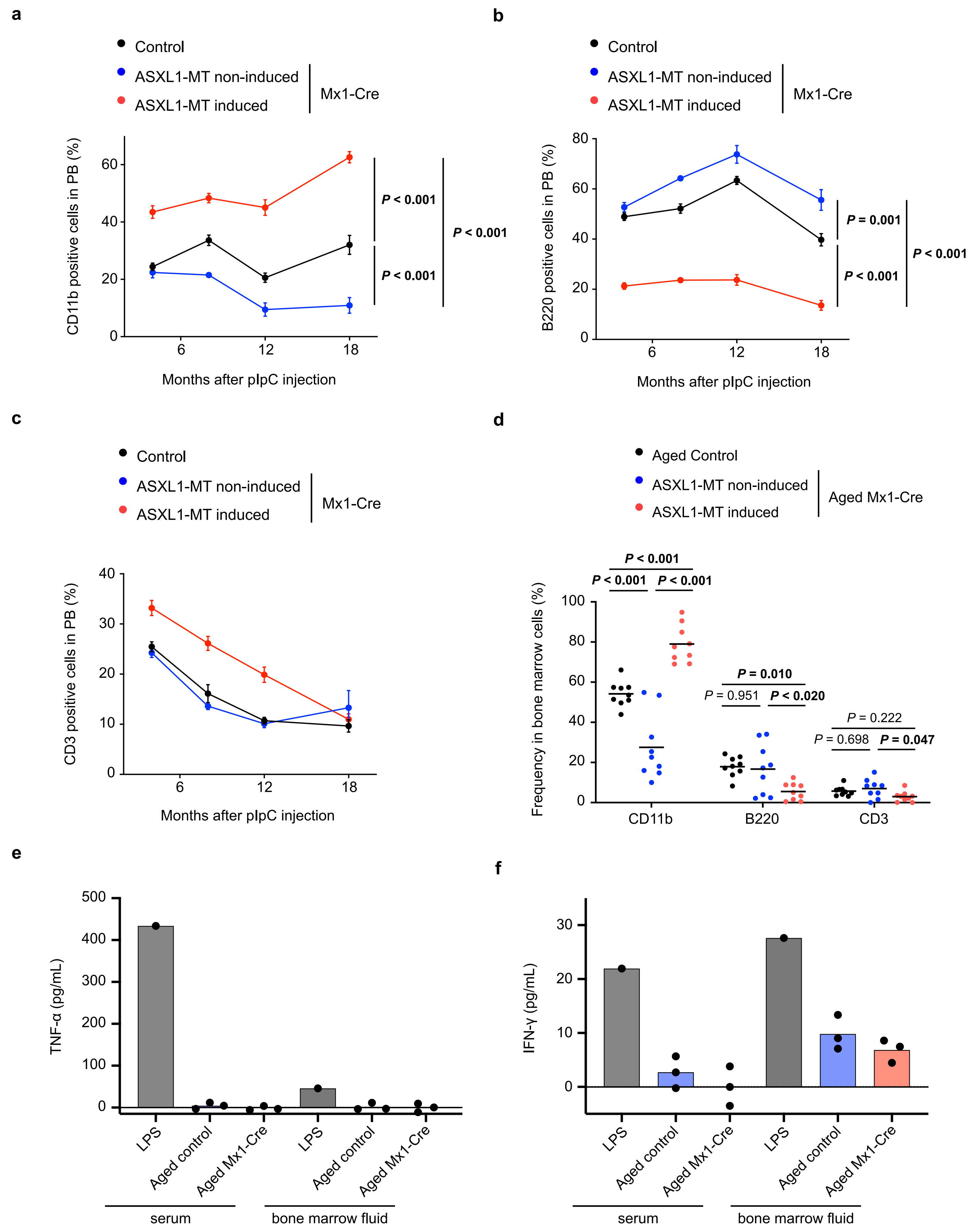

Supplementary Fig. 4

Supplementary Fig. 4 | ASXL1-MT confers a clonal advantage of LT-HSCs associated with skewed differentiation toward myeloid lineage. a-c, The frequency of CD11b (**a**), B220 (**b**), or CD3 (**c**) positive cells in peripheral blood at the indicated weeks after pIpC injections (n=20). Data are mean ± s.e.m. **d**, 18 months after pIpC injections, the frequency of CD11b, B220, and CD3 positive cells in bone marrow MNCs of aged control or *Mx1-Cre* ASXL1-MT KI mice are analyzed (n=9). **e,f,** Levels of pro-inflammatory cytokines including TNF- α (**e**) and IFN- γ (**f**) in serum or bone marrow fluid of aged *Mx1-Cre* ASXL1-MT KI mice (n=3). Data are assessed by one-way ANOVA with Tukey-Kramer's post-hoc test. *$P \leq 0.05$, **$P \leq 0.01$, ***$P \leq 0.001$

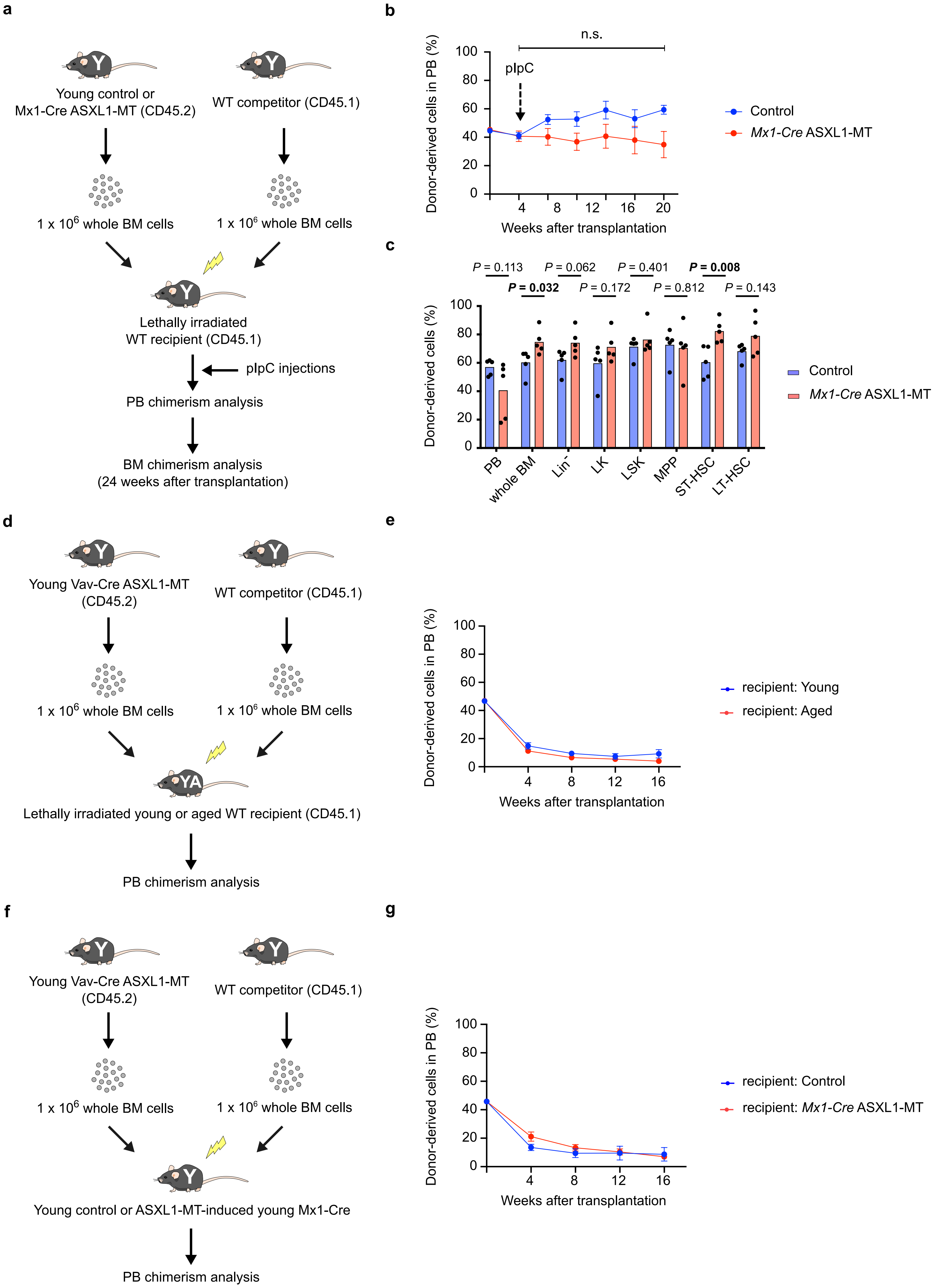

Supplementry Fig. 5

Supplementary Fig. 5 | Cell-intrinsic mechanisms are responsible for the defective repopulation potential of LT-HSCs in ASXL1-MT KI mice. a, The experimental design for competitive transplantations using *Mx1-Cre* ASXL1-MT KI mice as donors. 1×10^6 bone marrow MNCs isolated from young control and young *Mx1-Cre* ASXL1-MT KI mice were transplanted into lethally irradiated recipient mice. At 4 weeks after transplantation, recipient mice were intraperitoneally injected with pIpC (250 μg/mouse × 3 times). **b**, Levels of donor chimerism in peripheral blood were analyzed at the indicated weeks after transplantation (n=8). **c,** 24 weeks after transplantation, the frequency of donor-derived cells in peripheral blood (PB), whole bone marrow cells, Lin^- cells, LK cells, LSK cells, MPPs, ST-HSCs and LT-HSCs were analyzed (n=5). **d,** The experimental design for competitive transplantations using young or aged wild-type mice as recipients. 1×10^6 bone marrow MNCs isolated from young *Vav-Cre* ASXL1-MT KI mice were transplanted into lethally irradiated young or aged wild-type recipient mice. **e**, Levels of donor chimerism in peripheral blood were analyzed at the indicated weeks after transplantation (n=10 (Young) and 8 (Aged)). **f,** The experimental design for competitive transplantations using young *Mx1-Cre* ASXL1-MT KI mice as recipients. 1×10^6 bone marrow MNCs isolated from young *Vav-Cre* ASXL1-MT KI mice were transplanted into lethally irradiated young *Mx1-Cre* ASXL1-MT KI mice or young control mice that were intraperitoneally injected with pIpC (250 μg/mouse × 3 times) 4 weeks before transplantation. **g**, Levels of donor chimerism in peripheral blood were analyzed at the indicated weeks after transplantation (n=10). Data are mean ± s.e.m. *P≤0.05, **P≤0.01, two-tailed Student's *t*-test.

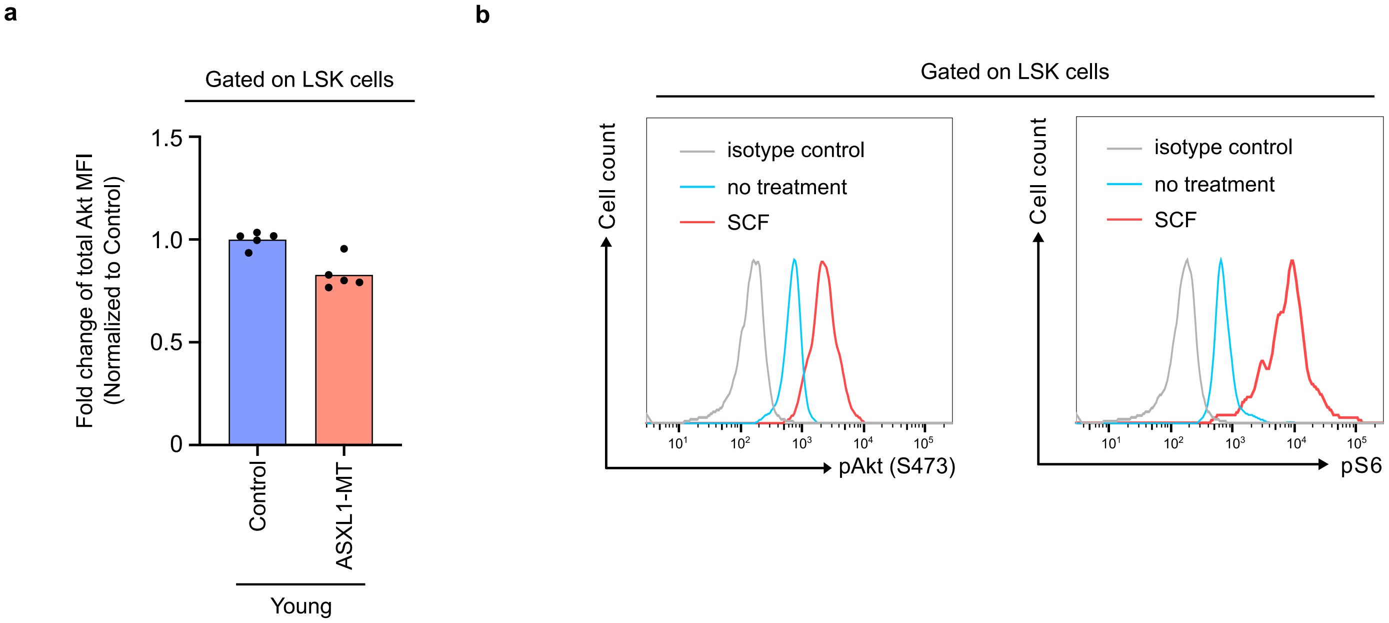

Supplementary Fig. 6

Supplementary Fig. 6 | Intracellular FACS analysis revealed increased phosphorylation of Akt without affecting the level of total Akt. a, The levels of total Akt in LSK cells of young control and young *Vav-Cre* ASXL1-MT KI mice (n=5). **b,** LSK cells isolated from wild-type mice were stimulated with SCF (10ng/mL) for 30 minutes, and were subsequently stained with an antibody against phosphorylated Akt (S473) (left panel) or phospho-S6 (right panel).

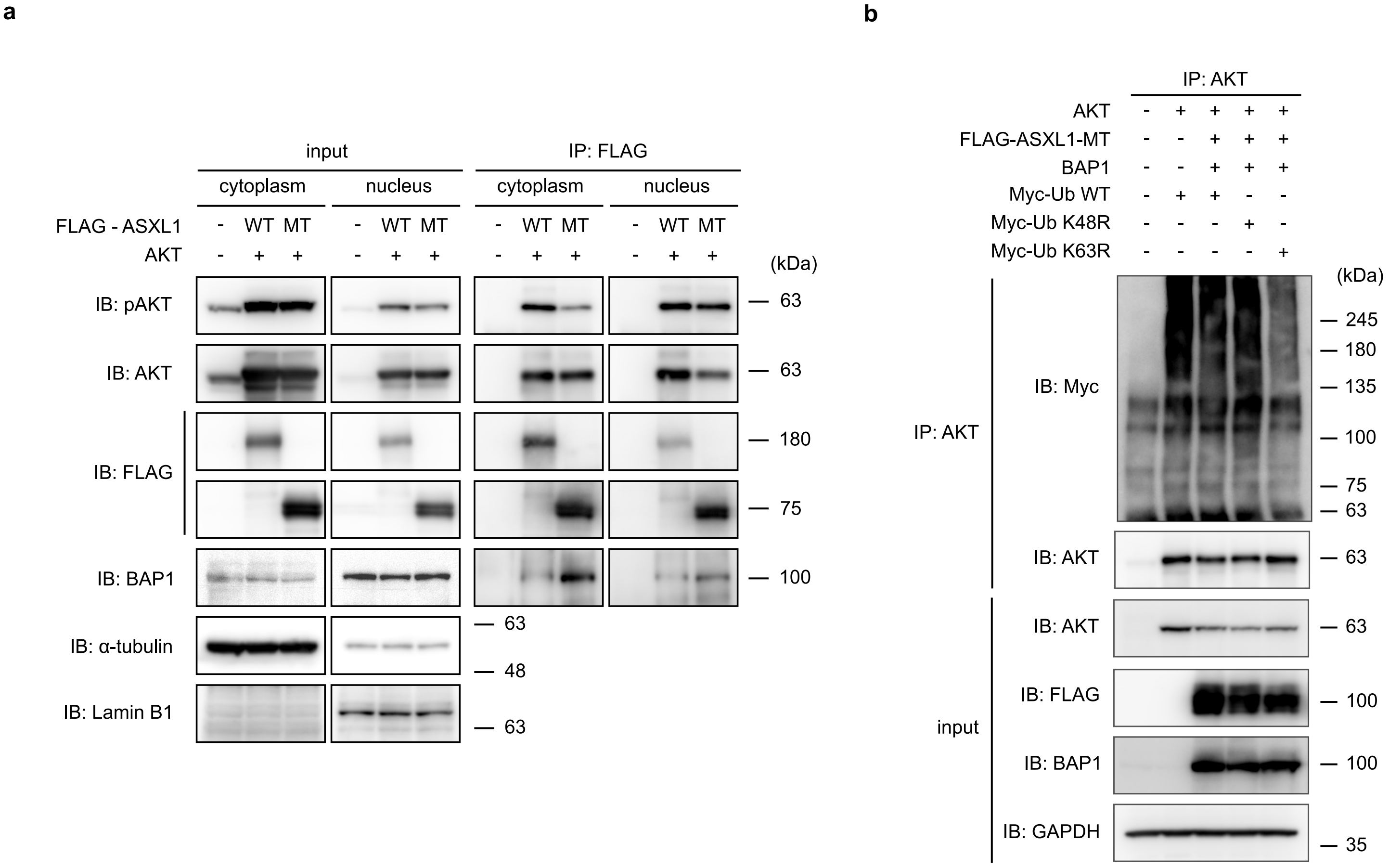

Supplementary Fig. 7

Supplementary Fig. 7 | ASXL1-MT combined with BAP1 to deubiquitinate K48-linked ubiquitination of AKT. a, 293T cells were transfected with AKT1, FLAG-ASXL1-WT, and FLAG-ASXL1-MT expressing vectors. Cytoplasm and nuclear fractions were isolated from whole cell lysate and were subsequently subjected to immunoprecipitation using anti-FLAG antibody followed by immunoblotting. **b,** 293T cells were transfected with AKT1, FLAG-ASXL1-MT, BAP1, Myc-Ubiquitin WT, Myc-Ubiquitin K48R, and Myc-Ubiquitin K63R expressing vectors. Total cell lysates were subjected to immunoprecipitation using anti-AKT antibody followed by immunoblotting. A representative experiment from at least n = 2 independent experiments is shown.

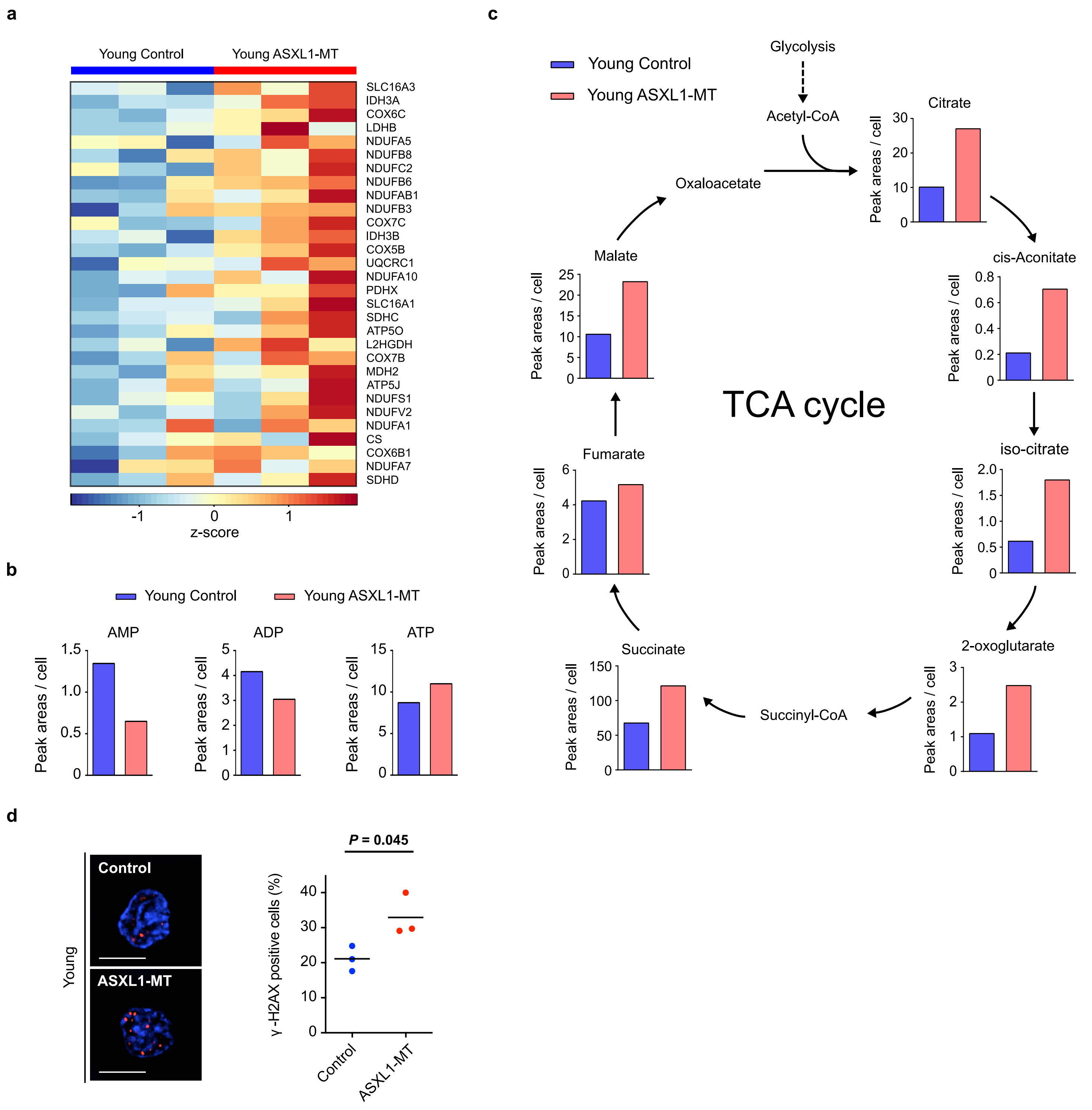

Supplementary Fig. 8

Supplementary Fig. 8 | ASXL1-MT alters mitochondrial dynamics and increases ROS-mediated DNA damage. a, Heat map of the top 30 up-regulated mitochondrial genes in LSK cells of young *Vav-Cre* ASXL1-MT KI mice (n=3). **b,c,** IC-MS quantification of the adenosine phosphate (**b**) and TCA cycle-intermediates (**c**) from pooled 5×10^4 LSK cells of young *Vav-Cre* ASXL1-MT KI mice. Peak areas are normalized to cell number. Cells were obtained from 3 animals per genotype. **d,** Representative images (Scale bars, 5 μm) (left panel) and percentage of LSK cells displaying γ-H2AX foci (right panel) (n=3). Statistical significance was assessed by two-tailed Student's t-test. *P≤0.05.

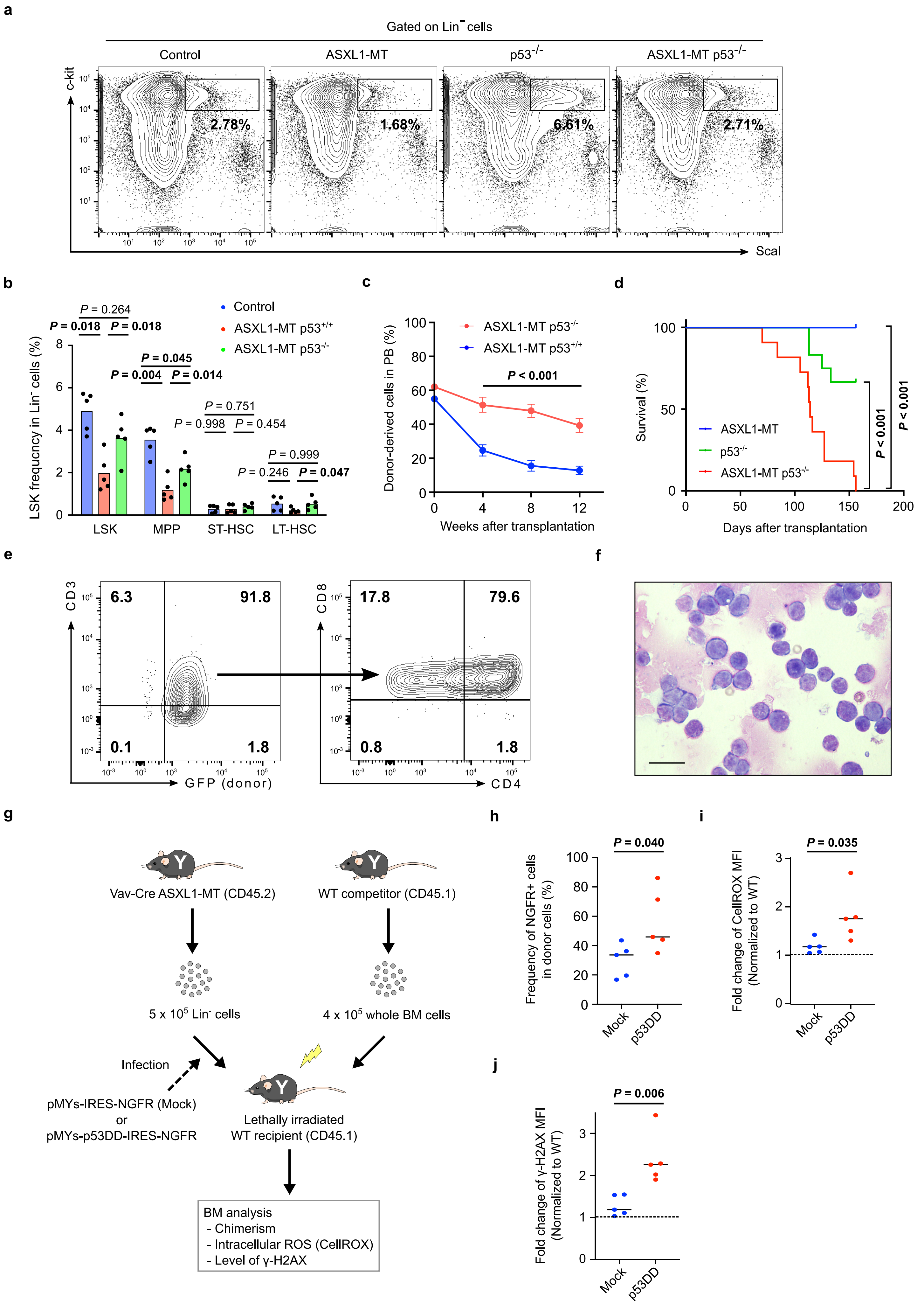

Supplementary Fig. 9

Supplementary Fig. 9 | Combined mutations of *ASXL1* and *p53* lead to the early development of thymic lymphoma. a, Flow cytometry profiles of wild-type, ASXL1-MT, ASXL1-MT/*p53*$^{-/-}$, and *p53*$^{-/-}$ mice are shown. **b**, The frequency of LSK population in Lin^{-} cells (n=5). **c,d,** Whole bone marrow cells isolated from ASXL1-MT, ASXL1-MT/*p53*$^{-/-}$ or *p53*$^{-/-}$ mice were transplanted into lethally irradiated recipient mice with competitor cells (n=7(ASXL1-MT), 11(ASXL1-MT/*p53*$^{-/-}$) or 12(*p53*$^{-/-}$)). Levels of donor chimerism in peripheral blood were analyzed at the indicated weeks (**c**). Kaplan-Meier survival curves (**d**). **e,f,** Representative flow cytometry profiles (**e**) and histology (**f**) of the thymus in mice transplanted with ASXL1-MT KI/*p53*$^{-/-}$ cells. **g,** The experimental design for transplantations using Lin- cells transduced with dominant-negative form of p53 (p53DD). Lin- cells from young *Vav-Cre* ASXL1-MT KI mice transduced with control or p53DD retrovirus were transplanted into lethally irradiated recipient mice. **h,** 4 weeks after transplantation, the frequency of p53DD-expressing cells in LSK fraction was analyzed (n=5). **i,j**, Effects of p53DD on ROS (**i**) and γ-H2AX (**j**) levels in LSK cells were determined (n=5). Data are mean ± s.d. Statistical significances are assessed by one-way ANOVA with Tukey-Kramer's post-hoc test (**b**), two-tailed Student's *t*-test (**c,h-j**) or log-ranked test (**d**). $^{*}P\leq0.05$, $^{**}P\leq0.01$.

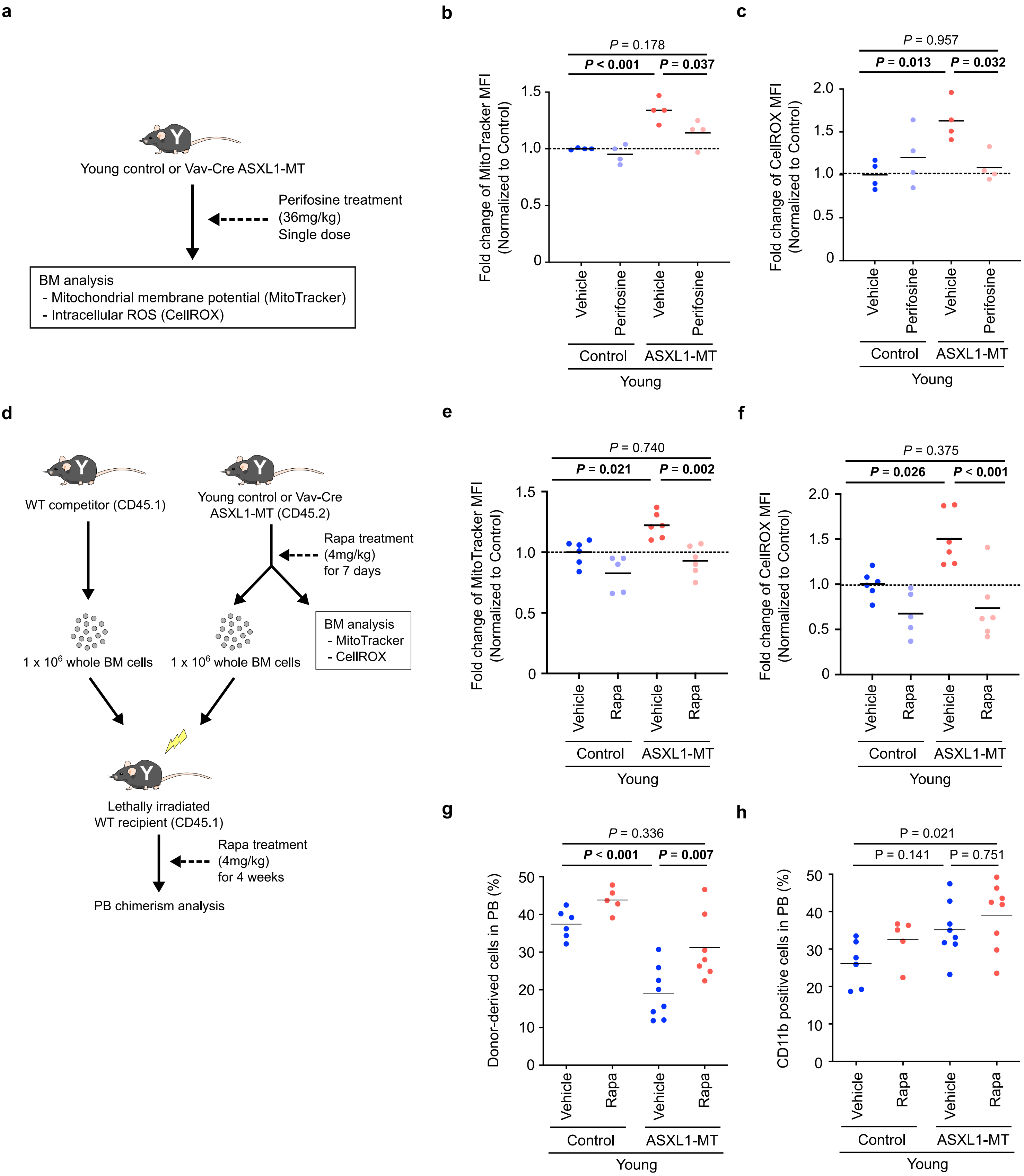

Supplementary Fig. 10

Supplementary Fig. 10 | The enhanced Akt/mTOR signaling caused by ASXL1-MT activates mitochondrial metabolism, increases ROS levels, and provokes dysfunction of HSPCs. a, Young control and young *Vav-Cre* ASXL1-MT KI mice were treated with a single oral dose of perifosine (36 mg/kg) intraperitoneally. **b,c,** 16 hours after perifosine administration, mitochondrial membrane potential (**b**) and intracellular ROS levels (**c**) in LSK cells were analyzed (n=4). **d,** The experimental design for treatment with rapamycin. Young control and young *Vav-Cre* ASXL1-MT KI mice were treated with rapamycin (4 mg/kg/day) intraperitoneally for 7 consecutive days. Whole bone marrow cells that had been treated with rapamycin were transplanted into lethally irradiated recipient mice with competitor cells. Recipient mice were continuously treated with rapamycin after transplantation. **e,f,** At the end of rapamycin administrations, mitochondrial membrane potential (**e**) and intracellular ROS levels (**f**) in LSK cells were analyzed (n=6 (Control-Vehicle), 5 (Control-Rapamycin), 6 (ASXL1-MT-Vehicle) and 6 (ASXL1-MT-Rapamycin)). Results are expressed as fold change relative to vehicle control group. **g,h,** 4 weeks after transplantation, the frequencies of donor-derived cells (**g**) and CD11b positive cells in donor-derived cells (**h**) in peripheral blood were analyzed (n=6 (Control-Vehicle), 5 (Control-Rapamycin), 7 (ASXL1-MT-Vehicle) and 7 (ASXL1-MT-Rapamycin)). Statistical significances are assessed by one-way ANOVA with Tukey-Kramer's post-hoc test. *$P \leq 0.05$, **$P \leq 0.01$, ***$P \leq 0.001$.

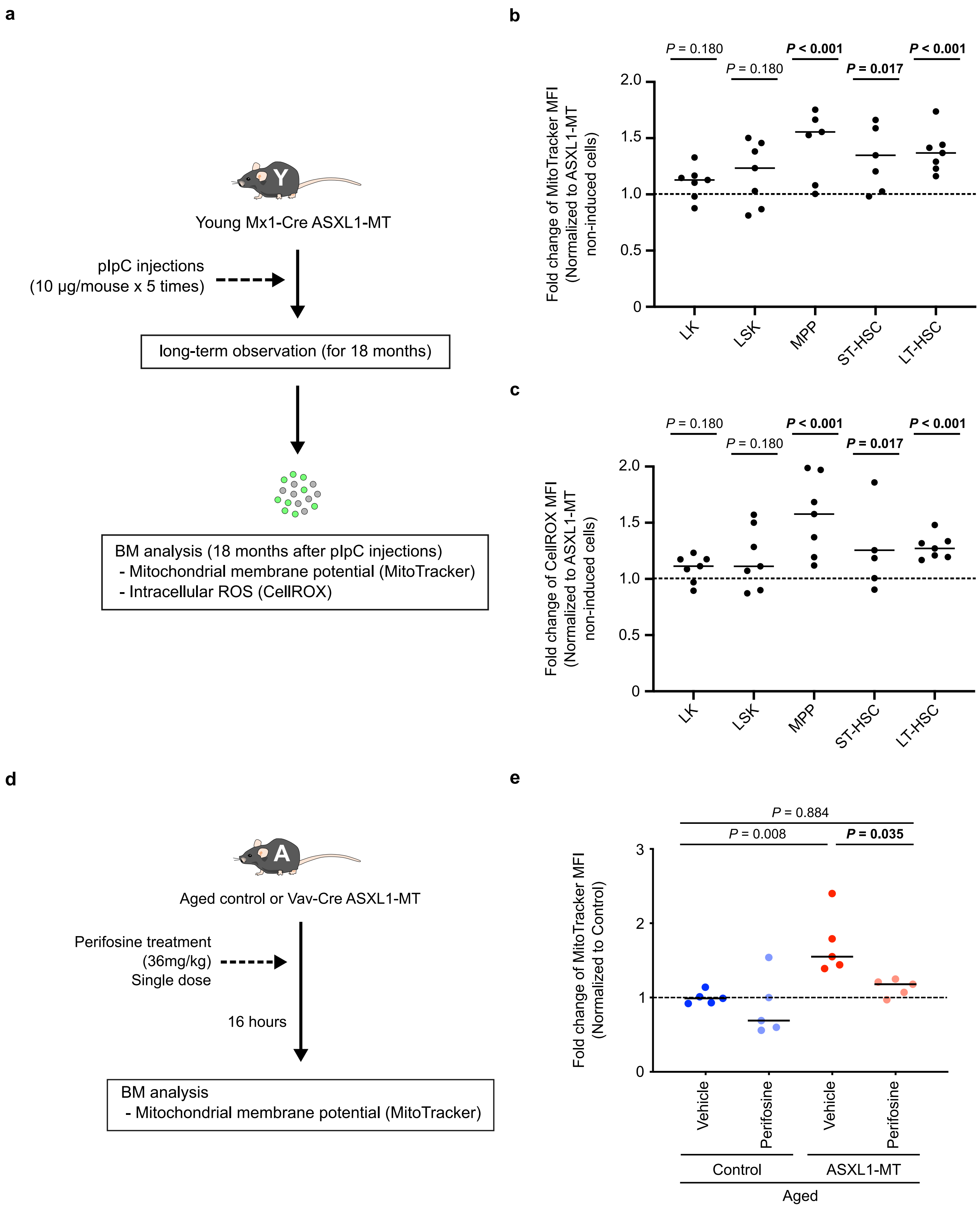

Supplementary Fig. 11

Supplementary Fig. 11 | ASXL1-MT provokes mitochondrial activation and increased ROS levels through activation of Akt in aged mice. a, The experimental design for partial induction of ASXL1-MT *in vivo* using *Mx1-Cre* ASXL1-MT KI mice (see Fig. 3). Young *Mx1-Cre* ASXL1-MT KI mice were partially induced to express ASXL1-MT by pIpC injections (10 μg/mouse × 5 times) at 12 weeks after birth. 18 months after pIpC injections, bone marrow cells of *Mx1-Cre* ASXL1-MT KI mice were analyzed. **b,c,** Mitochondrial membrane potential (**b**) and intracellular ROS levels (**c**) in LK cells, LSK cells, MPPs, ST-HSCs and LT-HSCs of ASXL1-MT-induced cells were analyzed (n=7). Results are expressed as fold change relative to their respective counterparts in ASXL1-MT non-induced cells. **d**, The experimental design for treatment with perifosine in aged mice. Aged *Vav-Cre* ASXL1-MT KI mice and age-matched control mice were treated with a single oral dose (36 mg/kg) of perifosine. **e**, 16 hours after administration, mitochondrial membrane potential of LT-HSCs was analyzed (n=5). Statistical significances are assessed by two-tailed Mann-Whitney's *u*-test (**b,c**) or one-way ANOVA with Tukey-Kramer's post-hoc test (**e**). $^*P \leq 0.05$, $^{**}P \leq 0.01$, $^{***}P \leq 0.001$.

Supplementary Table 1. Geneset enrichment analysis of LSK cells from young *Vav-Cre* ASXL1-MT-KI mice and littermate control mice.

Hallmark gene sets	NES	p-value	q-value
OXIDATIVE_PHOSPHORYLATION	1.91	0.000	0.001
HEME_METABOLISM	1.90	0.000	0.001
E2F_TARGETS	1.66	0.000	0.069
EPITHELIAL_MESENCHYMAL_TRANSITION	1.57	0.002	0.177
INTERFERON_GAMMA_RESPONSE	-1.35	0.012	0.103
INFLAMMATORY_RESPONSE	-1.36	0.016	0.113
IL2_STAT5_SIGNALING	-1.38	0.013	0.106
TGF_BETA_SIGNALING	-1.41	0.050	0.101
KRAS_SIGNALING_UP	-1.43	0.008	0.102
IL6_JAK_STAT3_SIGNALING	-1.58	0.002	0.041
ALLOGRAFT_REJECTION	-1.66	0.000	0.030
TNFA_SIGNALING_VIA_NFKB	-1.72	0.003	0.041

Geneset enrichment analysis (GSEA) to identify highly connected hallmark genesets in LSK cells from young *Vav-Cre* ASXL1-MT-KI mice. Statistically significant genesets (*p*-value<0.05 and *q*-value<0.25) are listed.

Supplementary Table 2. GSEA of CD34-positive bone marrow cells from MDS patients and healthy donors

AKT_UP.V1_UP	NES	p-value	FDR q-value
ASXL1 mutation	1.41	**0.009**	**0.145**
DNMT3A mutation	1.29	0.053	0.342
TET2 mutation	1.24	0.081	0.544
MTOR_UP.N4.V1_DN	NES	p-value	FDR q-value
ASXL1 mutation	-1.21	**0.011**	**0.216**
DNMT3A mutation	-1.34	**0.018**	**0.132**
TET2 mutation	-1.23	0.057	0.273

GSEA to compare gene expression in CD34-positive bone marrow cells from MDS patients harboring *DNMT3A* mutations (n=13), *TET2* mutations (n=33), or *ASXL1* mutations (n=21) to healthy control subjects (n=17) using genesets positively regulated by AKT signaling (AKT_UP.V1_UP) and negatively regulated by mTOR signaling (MTOR_UP.N4.V1_DN). *p*-value<0.05 and *q*-value<0.25 are considered statistically significant and are indicated in bold.
